# “*Candidatus* Similichlamydia laticola”, a Novel Chlamydia-like Agent of epitheliocystis in Seven Consecutive Cohorts of Farmed Australian Barramundi, *Lates calcarifer* (Bloch)

**DOI:** 10.1371/journal.pone.0082889

**Published:** 2013-12-03

**Authors:** Megan C. Stride, Adam Polkinghorne, Mark D. Powell, Barbara F. Nowak

**Affiliations:** 1 National Centre for Marine Conservation and Resource Sustainability, University of Tasmania, Launceston, Tasmania, Australia; 2 Institute of Health and Biomedical Innovation, Queensland University of Technology, Kelvin Grove, Queensland, Australia; 3 Norwegian Institute for Water Research, Trondheim, Norway; University of Lausanne, Switzerland

## Abstract

Six consecutively hatched cohorts and one cohort of pre-hatch eggs of farmed barramundi (*Lates calcarifer*) from south Australia were examined for *Chlamydia*-like organisms associated with epitheliocystis. To identify and characterise the bacteria, 59 gill samples and three pre-hatch egg samples were processed for histology, *in situ* hybridisation and 16S rRNA amplification, sequencing and comprehensive phylogenetic analysis. Cases of epitheliocystis were observed microscopically and characterised by membrane-enclosed basophilic cysts filled with a granular material that caused hypertrophy of the epithelial cells. *In situ* hybridisation with a Chlamydiales-specific probe lead to specific labelling of the epitheliocystis inclusions within the gill epithelium. Two distinct but closely related 16S rRNA chlamydial sequences were amplified from gill DNA across the seven cohorts, including from pre-hatch eggs. These genotype sequences were found to be novel, sharing 97.1 - 97.5% similarity to the next closest 16S rRNA sequence, *Ca*. Similichlamydia latridicola, from Australian striped trumpeter. Comprehensive phylogenetic analysis of these genotype sequences against representative members of the Chlamydiales order and against other epitheliocystis agents revealed these *Chlamydia*-like organisms to be novel and taxonomically placed them within the recently proposed genus *Ca*. Similichlamydia. Following Fredricks and Relman’s molecular postulates and based on these observations, we propose the epitheliocystis agents of barramundi to be known as “*Candidatus* Similichlamydia laticola” (sp. nov.).

## Introduction

Aquaculture is the fastest growing primary industry in Australia and, in 2011, a total of 75,188 tonnes of fish were produced with a value of almost one billion dollars to the Australian economy [1]. Established species such as the barramundi (*Lates calcarifer*) (Bloch, 1790), which was first developed in the mid-1980s and is now farmed in every state of Australia except for Tasmania, are strong contributors to this output. In 2011, barramundi aquaculture accounted for over 4,000 tonnes of product annually with a value of 36 million dollars [1]. Although barramundi aquaculture is well-established, an increased incidence of viral, bacterial and parasitic diseases occurs within the higher densities of farmed populations [2]. 

Epitheliocystis is a condition of the gills and skin of finfish and is caused by intracellular Gram-negative bacteria. It occurs in both wild and farmed fish populations and is currently known to affect over 80 different species of marine and freshwater fish [3,4], including barramundi [5,6]. Epitheliocystis is usually associated with Chlamydia-like organisms (CLOs) [3,4,7,8], which can cause epithelial hyperplasia, hypertrophy and inflammation of the infected tissue [5,9]. The identification of *Chlamydia*-like bacteria in association with epitheliocystis is increasing, with several new reports of new *Candidatus* species being described, namely *Ca*. Piscichlamydia cyprinis from grass carp (*Ctenopharyngodon idella*) [10], *Ca*. Parilichlamydia carangidicola from yellowtail kingfish (*Seriola lalandi*) [4] and *Ca*. Similichlamydia latridicola from striped trumpeter (*Latris lineata*) [11]. 

Epitheliocystis was previously described in the barramundi and the aetiological agent was thought to be a unique chlamydia, however, identification of this agent was limited to the amplification and sequencing of short 16S rDNA sequences from fixed tissue specimens [5,6]. The prevalence and factors associated with infections by this or other aetiological agents of epitheliocystis in barramundi are otherwise unknown. 

In the current study, we have performed a cross-sectional survey of six consecutively hatched cohorts of barramundi, plus pre-hatch eggs, for the presence and effects of epitheliocystis infections in a barramundi aquaculture facility. Fredricks and Relman’s molecular postulates were used with detailed histopathology, *in situ* hybridisation (ISH), near-full length 16S rRNA gene sequences and complete phylogenetic analyses as evidence for disease causation [12]. 

## Materials & Methods

### Ethics Statement

The samples were collected as a part of routine farm health monitoring and provided to the researchers as fixed samples. As such they were exempt from Ethics approvals (confirmed by University of Tasmania Ethics Committee).

### Sample collection

**Figure 1:**
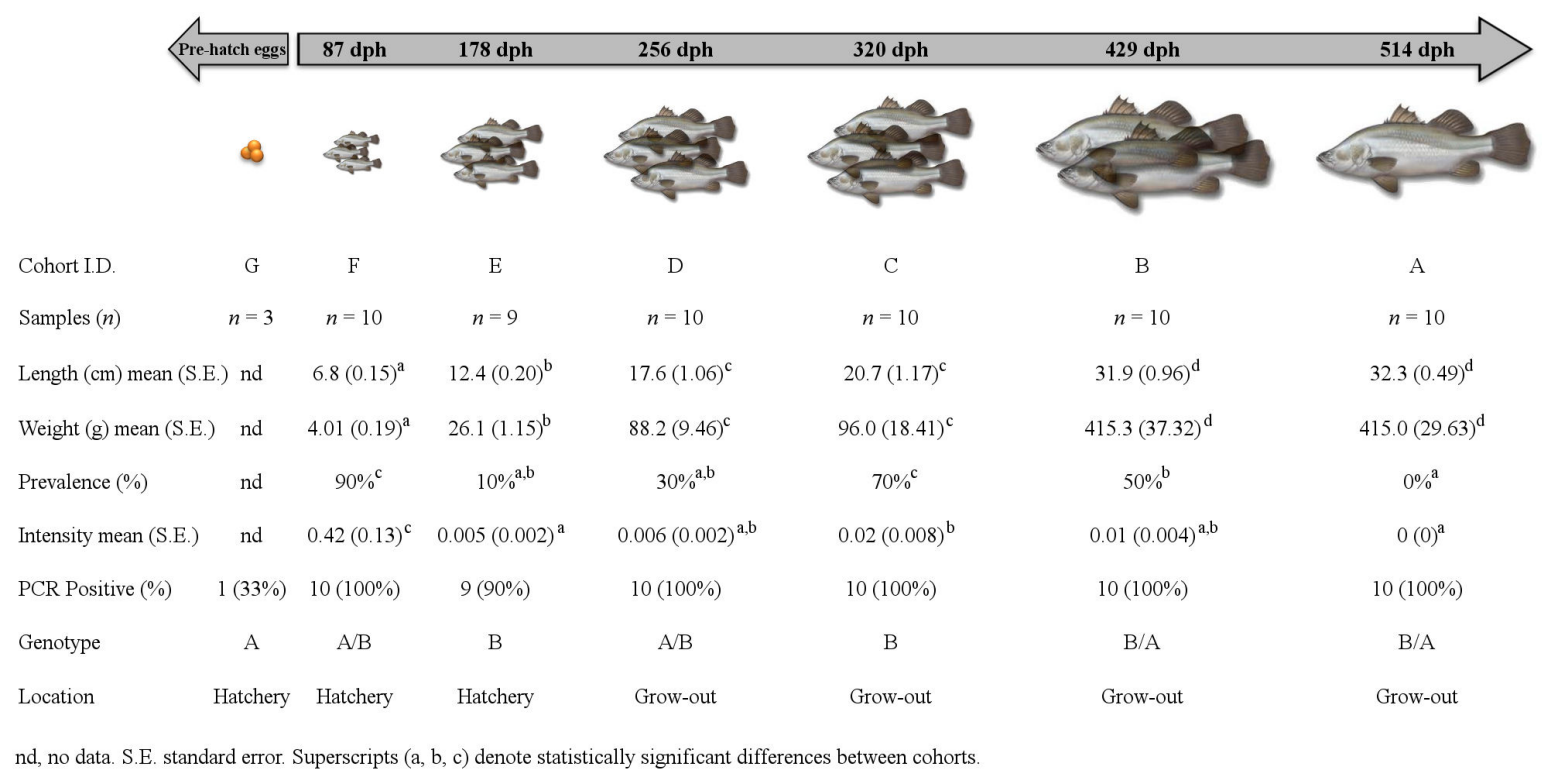
Timeline figure for each cohort of barramundi. Mean length (cm +/- SE), mean weight (g +/- SE), epitheliocystis prevalence (%), mean intensity (cysts/filament +/- SE), PCR positive (%), bacterial genotype and location of samples is shown. Each cohort has been designated a unique cohort I.D. (A-G inclusive).

Barramundi were spawned and reared at a commercial aquaculture facility in South Australia. Fish less than 100 mm in total length were raised in 1 m^3^ cages or 2 m^3^ flow-through tanks supplied with brackish water at the hatchery facility (cohorts E-G, inclusive, Figure 1). At 100-110 mm length, fish were transferred to the freshwater grow-out facility, where they were maintained in 50 m^3^ circular flow-through tanks (5,000-10,000 fish per tank depending upon size) supplied with geothermal artesian freshwater at 28°C and constant aeration (cohorts A-D, inclusive, Figure 1). Fish were fed twice daily to satiation using a commercial pelleted feed. 

A total of 62 samples from seven consecutive cohorts were taken during the winter of 2012. Ten fish from each of the cohorts A-D (inclusive) and cohort F, nine fish from cohort E and an additional three samples from a seventh cohort (cohort G) that was fertilised but yet to hatch were sampled. Total length and weight measurements were taken prior to the second gill arch on the sinistral side being sub-sampled into 10% neutral buffered formalin and a nucleic acid preservation solution (NAPS, 4 M ammonium sulphate, 25 mM sodium citrate, 10 mM EDTA; pH 5.5) as previously described [13] for analyses. A unique identifier has been given to each cohort to assist with descriptions (cohort A-G, inclusive, Figure 1).

### Histopathology

Formalin-fixed gills were trimmed and routinely processed for histology. Paraffin-embedded gills were sectioned at 5 μm and stained with haematoxylin and eosin. Sections were examined using light microscopy to identify epitheliocystis inclusions and associated lesions [4].

### DNA extraction, 16S rRNA amplification and sequencing

DNA was extracted from gill samples stored in NAPS using an optimised protocol for the Epicentre MasterPure^TM^ Complete DNA and RNA Purification Kit (Epicentre Biotechnologies, Madison, USA) [4]. A broad order Chlamydiales PCR assay targeting the 16S rRNA gene was performed to screen each sample for the presence of the Chlamydiales signature sequence. The Chlamydiales-specific primer pair 16SIGF and 16SIGR previously designed [14] and optimised [4] was used in a 50 µL reaction including three microlitres of extracted DNA. PCR amplification reaction and cycling conditions for these assays were as previously described [4]. The partial 16S rRNA sequence for the *Chlamydia*-like epitheliocystis agent was expanded with eubacterial reverse primers to 800 bp (16SIGF and 806R primer pair) and to a near full-length sequence (16SIG and 16SB1 primer pair). Cycling conditions, purification and sequencing was performed as previously described [4].

### In situ hybridisation

The *Chlamydia*-like organism within the epitheliocystis cysts was detected by ISH in 5 µm serial sections. Chlamydiales-specific antisense (DIG*ATG TA[T/C] TAC TAA CCC TTC CGC CAC TA*DIG) and sense (DIG*ATC CTA CGC TAC TAA GTC TCT CAT CA*DIG) DIG-labeled oligonucleotide probes as described were used [5,15] at a concentration of 10.2 pmol/mL [11]. *In situ* hybridization reaction details for this assay were as previously described [11].

### Molecular phylogenetic analysis

The 298 bp signature sequences of known *Chlamydia*-like epitheliocystis agents and the near full-length 16S rRNA regions sequenced here and data from representative Chlamydiales species and outgroup taxa obtained from GenBank were aligned and trimmed as previously described [4].

The software jModelTest version 0.1.1 [16] estimated TIM3+G and GTR+I+G as the best nucleotide substitution models for the signature sequence and near full-length datasets, respectively. Maximum likelihood (with 1,000 bootstraps) and Bayesian Inference (with 10,000,000 generations) analyses were performed using the software package MEGA5 [17] and Mr Bayes version 3.1.2 [18] run on the CIPRES portal [19] to explore relationships among these epitheliocystis taxa as previously described [4]. 

### Quantification

Following visual inspection of the H&E stained gill sections, the prevalence (expressed as a percentage) and intensity (intensity = cysts per section/filament per section) of cysts were calculated. The presence of CLOs detected by PCR was calculated as a prevalence (expressed as a percentage). Statistical analyses were conducted with the IBM SPSS Statistics package, version 20.0.0.1 (2011). Since the distribution of the data was non-normal (even after transformations), the non-parametric Kruskal-Wallis test with post-hoc comparisons and Mann-Whitney U test were used. A Spearman’s rank correlation test was used to identify if a significant relationship existed between fish length and the prevalence of epitheliocystis.

### Nucleotide sequence accession numbers

The 16S rRNA genotype sequences of the *L. calcarifer* epitheliocystis agents are available at GenBank under the accession numbers KF219613 and KF219614.

## Results

### Histopathology and prevalence of novel barramundi *Chlamydia-like* organism

**Figure 2:**
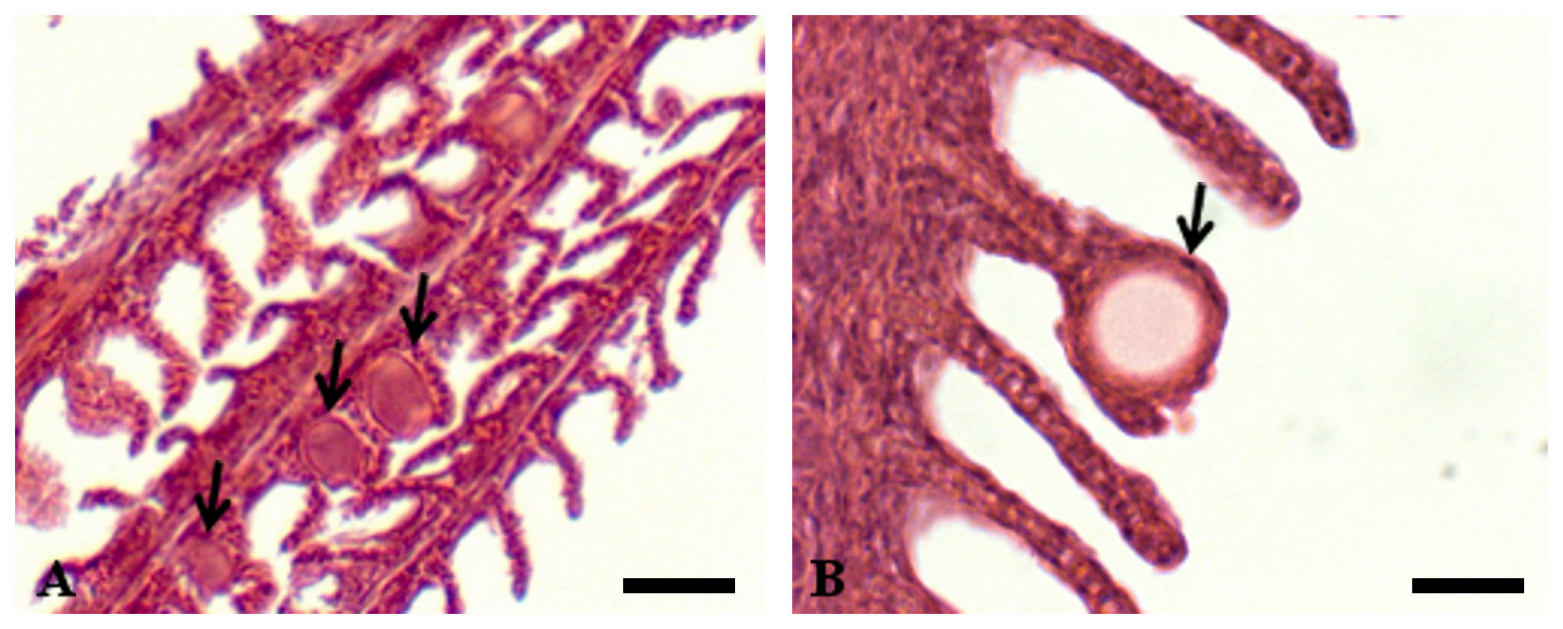
Epitheliocystis in gills of farmed Australian Barramundi (*Lates calcarifer*) stained with haematoxylin and eosin. (A) multiple membrane-enclosed basophilic granular epitheliocystis cysts (arrows) in the gills of hatchery sized barramundi (cohort F) (scale bar = 40 µm); (B) a single membrane-enclosed cyst (arrow) with epithelial hyperplasia around the cyst and at the base of the gill filament of barramundi 320 dph (cohort C) (scale bar – 25 µm). Images are representative of host reaction across the cohorts.

Mean length, mean weight, cyst prevalence (%) and intensity for each cohort are summarised in Figure 1. The age as days post hatch (dph) and origin of the fish sampled are also provided. There was a significant difference of fish length (KW = 50.704, df = 5, p < 0.001) and fish weight (KW = 50.694, df = 5, p < 0.001) between the cohorts (Figure 1). Epitheliocystis was present in all cohorts with the exception of cohort A, with prevalence as seen in histology ranging from 0 - 90% and intensity ranging from 0 - 0.42 (± 0.13) cysts/filament. There was a significant difference in prevalence (KW = 31.985, df = 5, p < 0.001) and intensity (KW = 30.519, df = 5, p < 0.001) between the cohorts (Figure 1), with the greatest prevalence and intensity in fish from cohort F (Figure 1). Epitheliocystis was seen in all cohorts of fish except for cohort A, which were the oldest fish sampled at 514 dph. In the cohorts with epitheliocystis, hypertrophied epithelial cells were filled with membrane-enclosed cysts containing basophilic material. Clusters of cysts were seen in samples from cohort F, which were the youngest fish sampled at 87 dph (Figure 2A). These cysts were found at the base of the secondary lamellae. Cysts were also seen in association with a proliferative epithelial cellular host response (Figure 2B).

### Molecular identification and phylogenetic analysis of novel CLOs

Initial screening by the 16S rRNA Chlamydiales-specific PCR assay showed that 95% (total samples tested, n = 62) of the barramundi samples were PCR positive for chlamydial DNA (Figure 1). Near full-length sequences were obtained from at least three samples per cohort (from both strands), with the exception of the pre-hatch eggs (one sample only). All PCR products were sequenced in both directions. Pairwise alignments of all near full-length sequences obtained across the cohorts revealed two distinct genotypes with 97.9% nucleotide sequence similarity to each other. A total of 29 single nucleotide polymorphisms (SNPs) were present between the two near full-length genotype sequences (1407 bp), occurring within the variable regions of the 16S rRNA gene. The SNPs were consistently found at the same positions from multiple samples within the gene when using the different primer pairs. Both genotypes were sequenced from fish of marine (cohorts E-G) and freshwater (cohorts A-D) origin (Figure 1). 

Barramundi CLOs sequences were compared against those in the NCBI database using the BLAST-n algorithm and revealed these sequences to be novel, sharing 97.1 - 97.5% similarity to the next closest 16S rRNA sequence, *Ca*. Similichlamydia latridicola, from Australian striped trumpeter [11]. 

**Figure 3:**
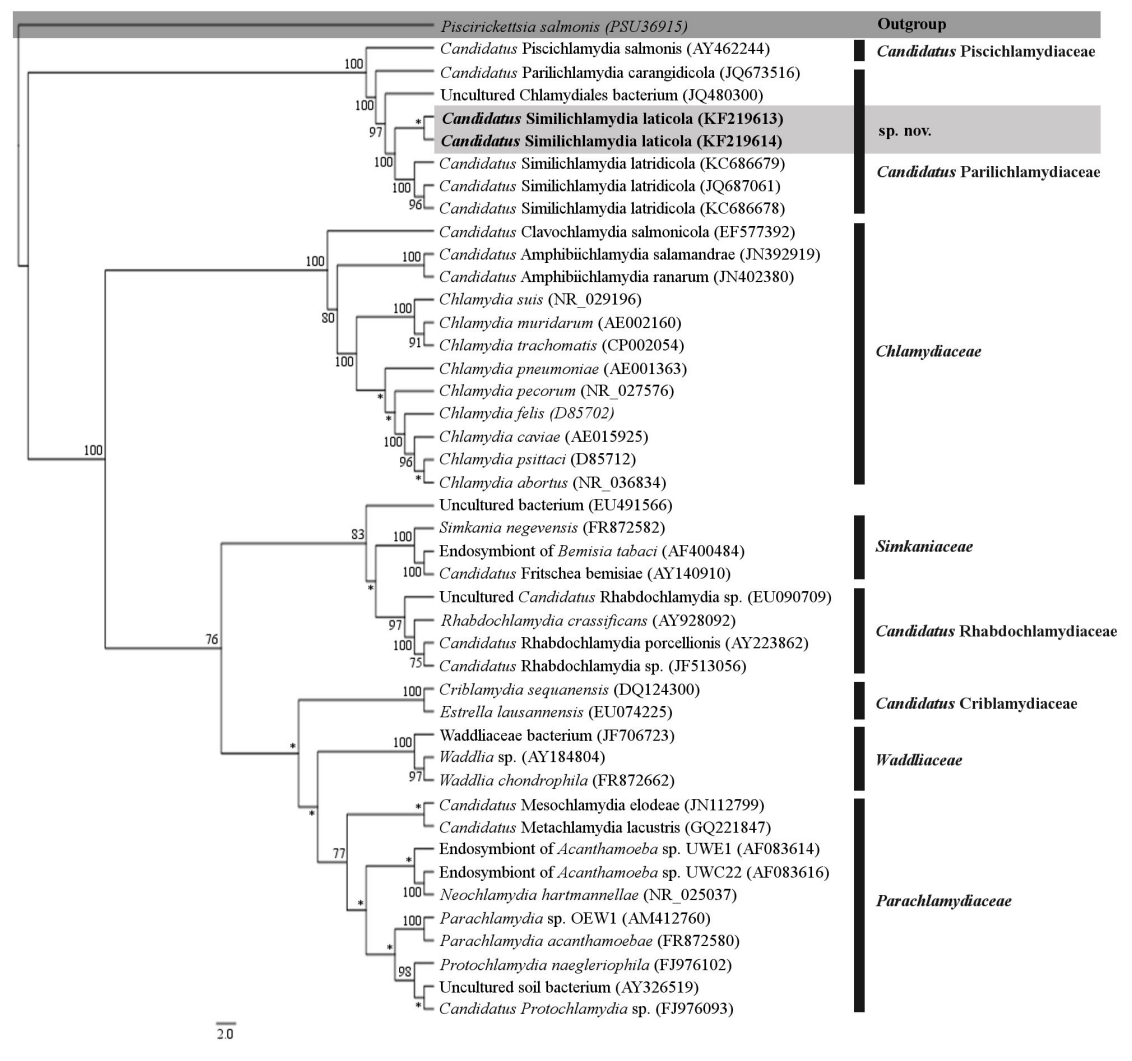
Phylogenetic relationship of ‘*Candidatus* Similichlamydia laticola’ detected in barramundi gills with known Chlamydiales taxa. Branching tree based on Maximum Likelihood analysis (MEGA5) of the 16S rRNA dataset. Bootstrap support values are given at the nodes, values less that 70% are indicated by an asterisk.

**Figure 4:**
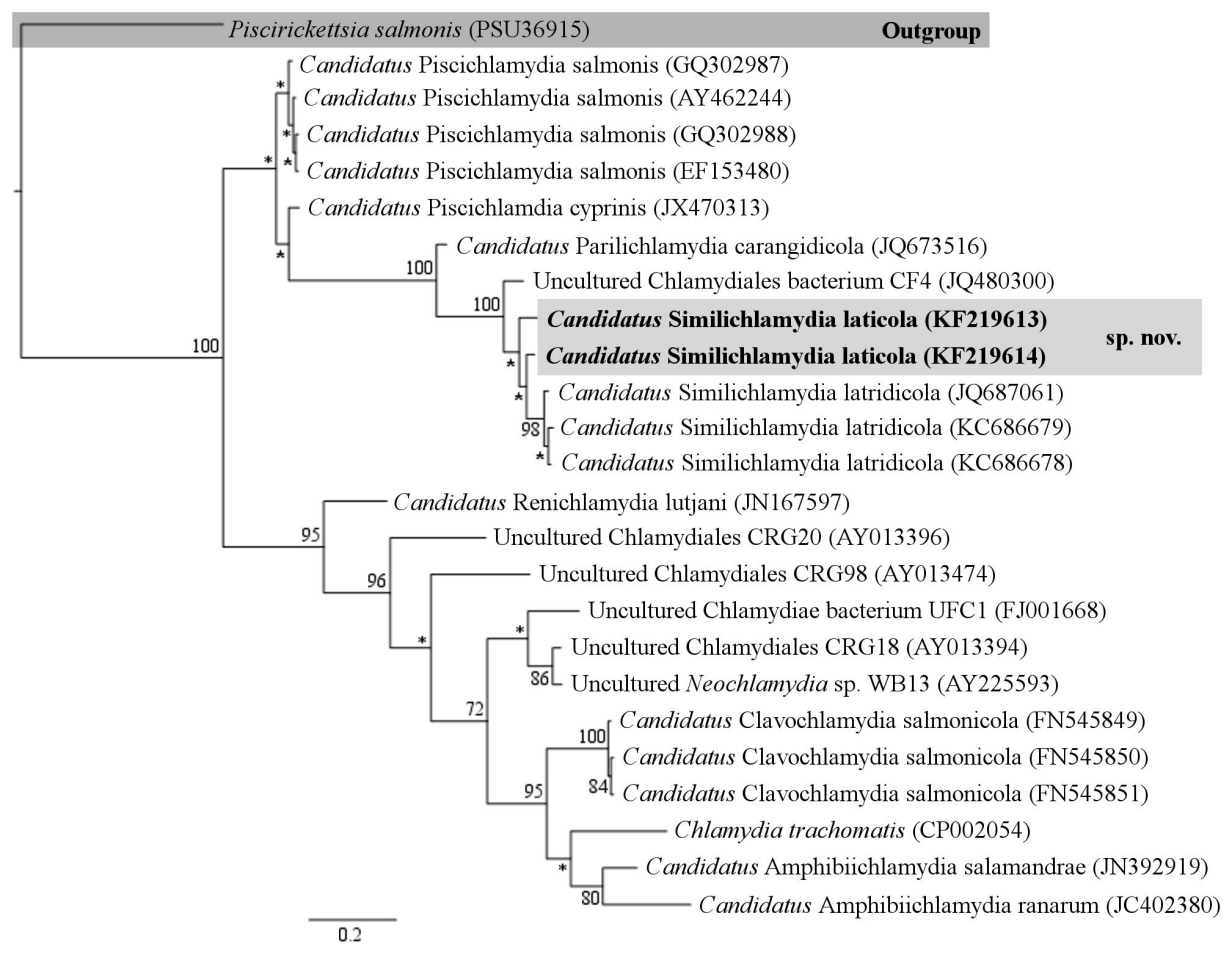
Phylogenetic relationships of the 16S rRNA signature sequences of known epitheliocystis agents. Bayesian Inference Analysis of the 16S rRNA signature sequence dataset was performed using the CIPRES portal (www.phylo.org/portal2/) [29]. Posterior probabilities are given at the nodes, with values less than 70% indicated by an asterisk.

Maximum likelihood analysis of the near full-length sequence dataset yielded 1,287 characters of analysis, which resulted in a well-supported phylogram with all of the currently recognised and candidate families within the Chlamydiales (Figure 3). Bayesian inference analysis of the signature sequence dataset of known epitheliocystis agents showed that the novel CLOs reported here from barramundi grouped together in a strongly supported clade with other members of the family *Ca*. Parilichlamydiaceae and in the same genus as epitheliocystis agents of striped trumpeter, *Ca*. Similichlamhydia (Figure 4). Phylogenetic comparisons between the sequences reported here and known epitheliocystis 16S rRNA sequences from GenBank confirm the novel species lineage of the CLOs reported here. 

#### In situ hybridisation

**Figure 5:**
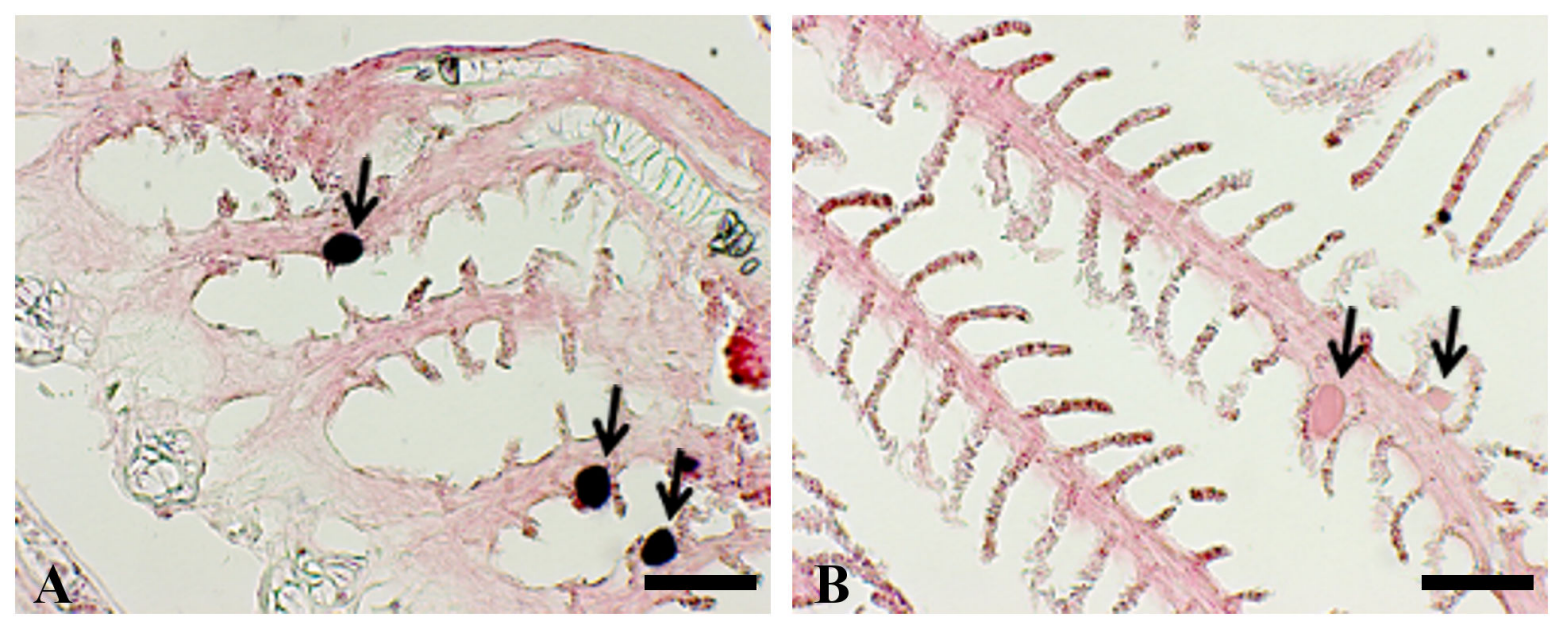
Detection of Chlamydiales bacteria in epitheliocystis infected gills by *in* *situ* hybridisation (ISH). Sections of cultured barramundi *Lates calcarifer* gill tissue tested with ISH; (A) positive reaction of epitheliocystis cysts in the gill with the antisense ISH probe, showing the purple/black colouration of the cysts (cohort F) (scale bar = 50 µm); (B) no reaction of epitheliocystis cysts in the gill with the sense ISH probe (cohort C) (scale bar = 50 µm). The images shown are representative of the staining found in other cohorts.

The presence of CLOs in the barramundi gills was confirmed by ISH using the Chlamydiales-specific probes. The application of the Chlamydiales-specific antisense probe lead to an intense and specific labelling of the epitheliocystis inclusions within the gill epithelium (Figure 5A). Sections receiving the sense probe showed no signal (Figure 5B).

## Discussion

In the current study, we have used several molecular methods to confirm that the agent of epitheliocystis found in barramundi gills farmed in South Australia is a novel member of the Chlamydiales. Phylogenetic analysis of the 16S rRNA gene showed the novel bacteria (two genotypes) from barramundi to be 97.5% similar to its nearest relative, the newly described *Ca*. Similichlamydia latridicola from striped trumpeter [11]. In addition, the novel bacterium is a new species of the *Ca*. Similichlamydia genus, as per the guidelines for classification of bacteria within the order Chlamydiales [14]. 

Barramundi was the first Australian farmed species to be reported with epitheliocystis [6]. Since this report, four other aquaculture species from Australia have been reported to be affected by epitheliocystis, yellowtail kingfish [20], silver perch (*Bidyanus bidyanus*) [21], Atlantic salmon (*Salmo salar*) [3] and striped trumpeter [22]. While there have been no other species of fish from the family Latridae reported to be affected with epitheliocystis, the condition has been reported from many species in the superfamily Percoidea, including yellowtail kingfish [4], jack mackerel (*Trachurus declivis*) [23], long-finned pike (*Dinolestes lewini*) [23], red sea bream (*Pagrus major*) [24] and silver perch [21], all of which have been reported from the Southern hemisphere.

The form of the epitheliocystis cysts observed in the barramundi of this study is in line with barramundi previously reported with this condition [6]. In the previous study, 12-week old barramundi fingerlings were observed to contain hypertrophied membrane-enclosed cysts filled with a basophilic material. This previous study did not report on older fish, however it has been reported in other fish species that older, larger fish have less of a host response to epitheliocystis infections than juveniles [25]. In addition, the discrepancy between negative (or low prevalence) histology and high positive PCR results has been reported previously in Atlantic salmon and yellowtail kingfish, indicating that PCR is a much more sensitive tool in detecting epitheliocystis [4,26]. The clustering of cysts observed here is similar to the epitheliocystis hyperinfection in striped trumpeter [22]. As further evidence that the epitheliocystis observed here is caused by CLOs, the order Chlamydiales-specific ISH probes clearly identified the *Chlamydia*-like bacteria within the epitheliocystis cysts *in situ*. The strong labelling reaction shown here is consistent with the results from both *Ca*. Piscichlamydia salmonis in farmed Atlantic salmon [27] and *Neochlamydia* sp. in farmed Arctic charr (*Salvelinus alpinus*) [28]. 

The detection of the CLOs from the pre-hatch eggs (cohort G) is a step forward in understanding the epidemiology and infection route of fish with epitheliocystis. There are two possible infection routes that can be determined; horizontal infection – where the infection source is coming from the environment; or vertical infection – with the infection being passed directly from the broodstock. As these fish have been maintained in captivity and were the 7^th^ generation broodstock, held within a partially recirculating system with limited water flow and input of filtered and U.V-treated water, it is reasonable to presume that horizontal transmission is unlikely. With the detection of the CLO from the pre-hatch eggs and the multiple genotypes of CLOs sequenced from fish of both marine and freshwater origins, vertical transmission as the route of infection is plausible in this case. More data are needed to prove this hypothesis, and the development of *in vitro* culturing methods will allow researchers to confirm the infection transmission route and answer what environmental factors cause a high infection level in fish populations.

Following the guidelines for classifying bacteria in the order Chlamydiales [14], the current study identified two closely related but distinct bacterial sequence genotypes of the barramundi CLOs across the cohorts and sites. These CLOs did not match a previously reported CLOs from farmed Australian barramundi [5]. The previously reported 298 bp signature sequence was obtained from a 14 year old archival tissue sample that was formalin-fixed and paraffin embedded. The sequence obtained was distinctly different to that reported in this study which may be due to the geographical difference of sample sources, but may also be associated with issues with the PCR amplification and sequencing of formalin-fixed and paraffin-embedded tissue samples from the previous study. 

In the absence of a viable *in vitro* CLO culturing method, this study has used the molecular postulates of Fredericks and Relman [12]. In summary, the combination of epitheliocystis diagnosed by standard histopathology, strong reactivity of cysts to Chlamydiales *in situ* probes and extensive phylogenetic analysis of gene sequence data from both near full-length sequences from representative Chlamydiales species and signature sequences from epitheliocystis agents of other fish species which have been identified across six consecutively hatched cohorts and pre-hatch eggs provides convincing evidence that the CLOs identified are the main epitheliocystis aetiological agent in farmed barramundi examined in the present study. Based on the novel 16S rRNA sequence genotypes, the percentage of sequence divergence from other Chlamydiales species and the observed phylogenetic relationships of the bacterial genotypes to other taxa within the order [14], the name ‘*Candidatus* Similichlamydia laticola’ (sp. nov.) (Order Chlamydiales) is proposed to identify this *Chlamydia*-like agent of farmed Australian barramundi. 

### Taxonomy

“*Candidatus* Similichlamydia laticola” sp. nov., recovered from barramundi (*Lates calcarifer*). Laticola sp. nov.; la.ti'co.la. N.L. n. Lates -is, a zoological genus name; L. suff. -cola (from L. n. incola), inhbitant, dweller; N.L. n. laticola, Latis-dweller, isolated from barramundi (*Lates calcarifer*).

Obligate intracellular bacteria infecting fish gills classified as Chlamydiales. The new species 16S rRNA sequence is differentiated from all formally recognised and Candidatus Chlamydiales taxa based on a combination of morphological and genetic differences. The new species is 2.1 - 2.9% different from the 16S rRNA sequence of *Ca*. Similichlamydia placing it within this genus, but not a member of the species taxon *latridicola*, according to the classification scheme of Everett [14]. Membrane-enclosed cysts present, staining basophilic under haematoxylin and eosin. Inclusions are not site specific and are found along the gill filament at the base, middle and tip of the lamellae. Inclusions react with an intense and specific labelling of the epitheliocystis inclusions when the ISH 16S rRNA Chlamydiales probe is applied. 
